# Insights into Wheat Genotype‒*Sphaerodes mycoparasitica* Interaction to Improve Crop Yield and Defence against *Fusarium graminearum*: An Integration of FHB Biocontrol in Canadian Wheat Breeding Programmes

**DOI:** 10.3390/pathogens13050372

**Published:** 2024-04-30

**Authors:** Antonia Powell, Seon Hwa Kim, Pierre Hucl, Vladimir Vujanovic

**Affiliations:** 1 Food and Bioproduct Sciences, University of Saskatchewan, Saskatoon, SK S7N 5A8, Canada; 2 Plant Sciences, Crop Development Centre, University of Saskatchewan, Saskatoon, SK S7N 5A8, Canada

**Keywords:** Fusarium head blight, wheat genotypes, resistance, biological control, mycoparasite, *Sphaerodes mycoparasitica*, bread wheat, durum wheat, *Fusarium graminearum*

## Abstract

Fusarium head blight (FHB) is a major threat to wheat crop production and food security worldwide. The creation of resistant wheat cultivars is an essential component of an integrated strategy against *Fusarium graminearum*, the primary aetiological agent that causes FHB. The results of this study show that the deployment of proto-cooperative interactions between wheat genotypes and mycoparasitic biocontrol agents (BCAs) can improve crop yield and plant resistance in controlling the devastating effects of FHB on wheat agronomic traits. A Fusarium-specific mycoparasite, *Sphaerodes mycoparasitica*, was found to be compatible with common and durum wheat hosts, thus allowing the efficient control of *F. graminearum* infection in plants. Four genotypes of wheat, two common wheat, and two durum wheat cultivars with varying FHB resistance levels were used in this greenhouse study. The BCA treatments decreased FHB symptoms in all four cultivars and improved the agronomic traits such as spike number, spike weight, seed weight, plant biomass, and plant height which are vital to grain yield. Conversely, the *F. graminearum* 3ADON chemotype treatment decreased the agronomic trait values by up to 44% across cultivars. Spike number, spike weight, and seed weight were the most improved traits by the BCA. A more measurable improvement in agronomic traits was observed in durum wheat cultivars compared to common wheat.

## 1. Introduction

The Food and Agriculture Organization (FAO) of the United Nations’ annual reports on wheat production state that approximately 776.7 million tonnes of wheat was produced in the year 2020–2021 [1]. For this same period, the United States Department of Agriculture (USDA) also reported that the consumption of wheat and wheat products far exceeded wheat production. To meet the rising demand for wheat and wheat products, it stands to reason that wheat yields have to be substantially increased. However, the estimated improvement in wheat production of 0.5–1% per year is below the 2.4% required to meet global demands [2,3]. In addition to the increased demand for this commodity, the UN projects that in the next three decades the world population will likely increase by 21% to roughly 10 billion people [4]. Therefore, to feed the world population, wheat production must further be increased to 5 tonnes/hectare to sustain the global population in the next three decades. Currently, the world average wheat yield is approximately 3 tonnes/ha [1]. Unfortunately, most of the wheat-producing countries have not been able to achieve ≥3 tonnes/ha (t ha*^−^*^1^), and still only a few of these countries have been able to achieve ≥5 t ha*^−^*^1^. Presently, Canada, for the last three years (2020–2023) has narrowly achieved yield levels ≥3 t ha^−1^ [5]. Low yield levels could be attributed to different variables such as climate conditions, cultivar potential, cultivar pest resistance, biotic and abiotic stressors, disease management, and agronomic practices. Given all these variables, Powell and Vujanovic [6] postulate that a devasting disease like FHB caused by the aggressive *Fusarium graminearum* 3ADON population, which decreases grain yield and crop quality, contaminates food via destructive toxins, and compromises food safety and security, is likely to worsen in the years to come. It is therefore of great importance that innovative methods are implemented to secure a sustainable food supply to mitigate the demands of an increasing world population.

It is generally accepted that breeding ”green revolution” efforts have led to the development of higher yielding varieties with improved agronomic traits, some of which have been highly beneficial in the wheat industry. Indeed, the release of semi-dwarf plants has significantly increased the grain yield in different crops including wheat. Norman Borlaug was awarded the Nobel Peace Prize (1970) for the contribution that he made to wheat development. His pioneering work included successfully transferring two semi-dwarf alleles *Rht-B1b* and *Rht-D1b* into common and durum wheat [7]. Since then, many high-yielding varieties, 90% or more in North America, possess one of these semi-dwarf alleles. However, these same alleles in dwarf plants that have been instrumental in increasing the grain yield are often associated with an increase in FHB severity. Hilton et al. [8] reported that semi-dwarf wheat plants by reason of reduced height are closer to the *Fusarium* inoculum source situated on the top of the soil/straw, and therefore increase the probability of successful entry into and infection in the florets of the spike. Consequently, having taller wheat plants is considered beneficial as a form of avoidance of FHB, while active protection can be eventually compensated through the BCA application on compatible plant hosts or crop cultivars.

Spike health is another essential component for crop productivity. The spike is the grain-bearing organ whose physical characteristics are proxy measures of grain yield, but, unfortunately, it is also one of the main targets of *Fusarium* pathogens. An infection in a spike has the potential to affect the spike number, spike weight, and more so the seed weight. In developing kernels, the extent of shrivelling or damage of the spikelet by *Fusarium* pathogens depends on when and where on the spike the infections occur under conducive weather conditions. Spike tissue differences and biochemical profilers were observed between the infected rachis of FHB-resistant and susceptible cultivars [9]. If *Fusarium* invades and kills the rachis, the main axis of the spike, the spikelets above that point containing the mature or developing grains will die, even if they are not colonized by the fungus [10]. This eventually results in no grain at all or small shrivelled grains that are usually lost during the threshing process. It is therefore essential that the wheat’s spikes are protected from *Fusarium* infection.

Improvement of agronomic traits in Canadian cereals is usually achieved by genetic gains through breeding [11]. Favorable germplasm for these traits is sought after, and when found after years of research they are used in creating better grain yielding varieties [12]. However, the difference in yield is often limited by the region, climatic conditions, agronomic factors, and the potential of the improved variety. It has been reported that the major wheat producing countries in the world over the last decades have undoubtedly taken advantage of genetic variation and have produced varieties with differing yield potential [13,14]. Even though breeding programs around the world have made these significant strides in optimising crop traits for greater yield, the progress is not nearly identical across all wheat varieties. In low-yielding areas, for instance, genetic crop improvements pale in comparison to high-yielding areas. Additionally, countries such as the USA, Chile, France, and Brazil have reportedly reached the maximum limit in wheat yields [15,16,17]. It is therefore imperative that alternative methods are found and investigated for their potential to improve, maintain, or supplement essential agronomic traits in wheat in the fight against FHB.

Plant endophytes have been used as biocontrols protecting their hosts from various threats [18] over the generations [19]. The endophytes shift wheat resistance and combat major diseases throughout the plant’s life cycle from a seed to a mature plant [20]. The endophytic biocontrol, *Sphaerodes mycoparasitica* Vujan. is a specific mycoparasite of phytopathogenic and mycotoxigenic *Fusarium* hosts [21] that has been reported to be particularly suitable for prenatal plant care for protecting flowers and germinating seeds from *Fusarium* infection and thus providing an early control of FHB [22] for crop establishment and higher yield [20,23]. In numerous studies, *S. mycoparasitica* has been shown to downregulate the expression of *Tri* (trichothecene) and AUS (aurofusarin) genes in fusaria and degrade mycotoxins such as DON, 3ADON, 15ADON, and ZEA [23,24]. Evidence from ongoing studies has also shown that *S. mycoparasitica* alone can bolster defence in wheat and barley against FHB and mycotoxins as well as in combination with low doses of synthetic chemicals, including tebuconazole (Folicur^®^) and prothioconazole plus tebuconazole (Prosaro^®^), a fungicide in both greenhouse and field experiments on small cereals [6].

The objectives of this study were to investigate the effects of this BCA (*S. mycoparasitica*) on the yield and major agronomic traits in both moderately resistant and moderately susceptible common wheat and durum wheat genotypes under high *Fusarium graminearum* pressure.

## 2. Results

### 2.1. Effects of BCA Treatments on Agronomic Traits 

**Figure 1:**
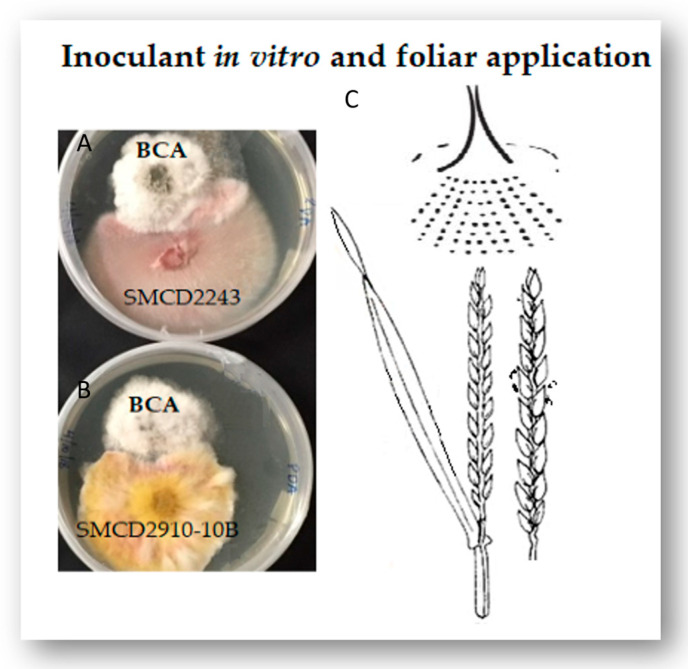
BCA mycoparasite application: Inoculant interaction on Potato Dextrose Agar (PDA) plates with (**A**) *F. graminearum* 3ADON SMCD2243 strain and (**B**) *F. graminearum* 3ADON SMCD2910–10B; (**C**) Inoculant spraying on wheat host plants during the development of the flowering stage (Feekes growth stage 10–10.1; Zadok 50–58) when plant sugars such as fructose, glucose, sucrose, and fructans are just starting to accumulate in the inflorescence.

**Figure 2:**
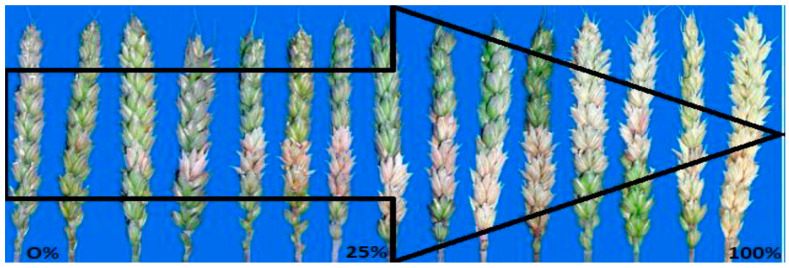
A visual scale to estimate the severity of Fusarium head blight of wheat. It is a modified scale to estimate the severity of Fusarium head blight in wheat. (Reviewed November 1988 N.D. State Univ. Ext. Publ. PP-1095; website: https://library.ndsu.edu/ir/handle/10365/9187) (accessed on 21 July 2019.).

The nine Fusarium-specific BCA treatments (Table 1) applied with and without fungicide to wheat and durum crops (Figure 1) induced changes in agronomic traits (plant growth and biomass, spike formation and seed yield) specific to each host genotype as an interactive mechanism of the first line of protocooperative defence against the invasive Fusarium (Figure 2) pathogen.

#### 2.1.1. Spike Number

**Figure 3:**
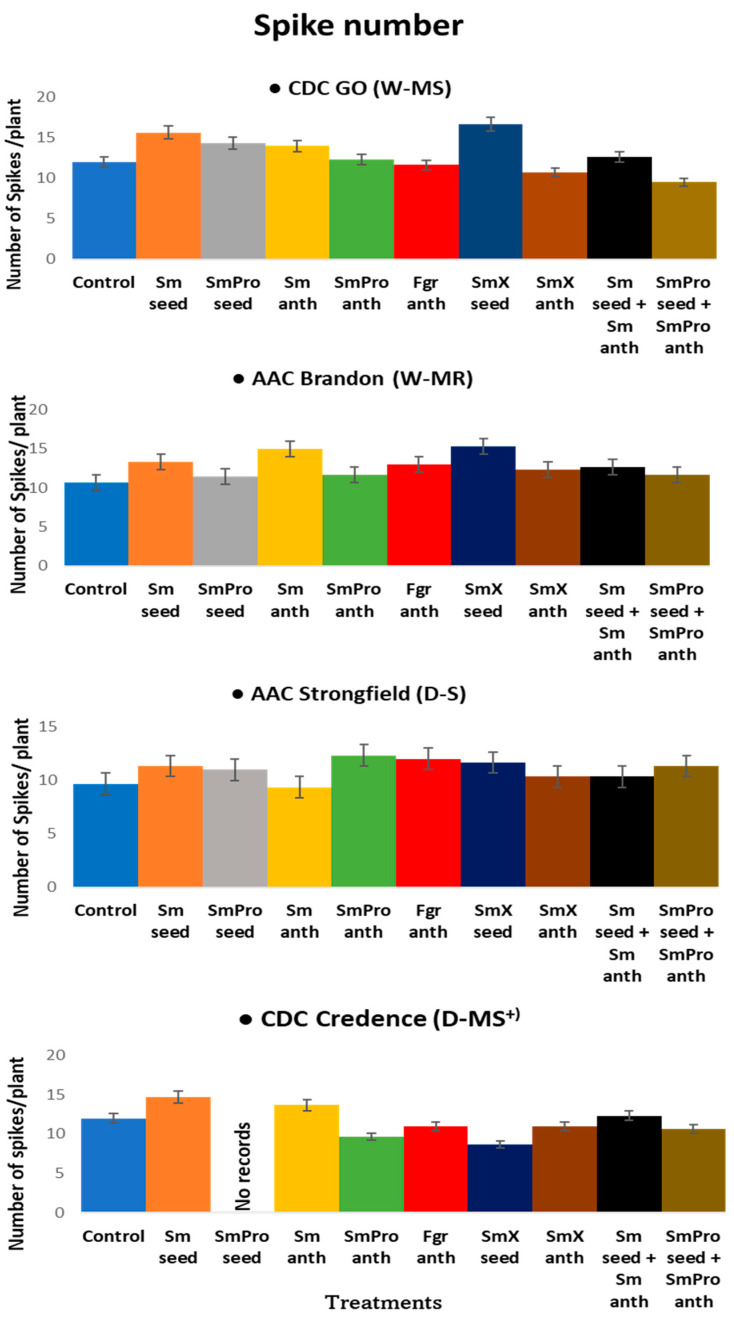
The effect of treatments on spike number of two common wheat cultivars—CDC Go moderately susceptible (W-MS) and AAC Brandon, moderately resistant (W-MR) and two durum wheat species—AAC Strongfield susceptible (D-S) and CDC Credence, intermediately/moderately resistant (D-MS^+^/D-MR) cultivars. The data for each cultivar were statistically analyzed using One-way Analysis of Variance (ANOVA) with Duncan’s Multiple Range Test (DMRT) at *p* = 0.05. The means and standard deviations are represented by error bars. All plants in all treatments except for the control and *Fgr_anth_* were inoculated with *Fusarium graminearum* within an 8 h window after the application of the BCA treatments. There were five plants per pot for each treatment in each cultivar. Each treatment for each cultivar had three replicates.

The average spike number per pot for each cultivar was Go—13 spikes/pot, Brandon—13 spikes/pot, Strongfield—11 spikes/pot, and Credence—10 spikes/pot (Figure 3). The overall highest spike number for a single pot was recorded for the wheat cultivars Go (W-MS) and Brandon (W-MR), i.e., 16 spikes each with treatment 7, *Sm*X_seed_ (*S. mycoparasitica* SMCD 2220-01 + SMCD 2220-02(5)—a mixture of BCA beneficial strains applied at the seed stage). Strongfield, the susceptible durum cultivar (D-S), had the highest spikes/pot with treatment 5, *Sm*Pro_anth_ (*S. mycoparasitica* 2220-01 + fungicide at anthesis), while for Credence, the intermediately/moderately resistant durum cultivar (D-MS^+^/D-MR), the highest spike number of 15 spikes for a single pot was observed with the treatment of *Sm*_seed_ (*S. mycoparasitica* 2220-01 applied to seeds) (Figure 3). The lowest spike number for Go (10 spikes for a single pot) and Credence (9 spikes for a single pot) was observed with treatment 7, *Sm*X_seed_ when compared to the control treatment. The lowest (12 spikes for a single pot) for Brandon was observed with the control and the lowest spike number of 9 for Strongfield was observed with *Sm*_anth_ (*S. mycoparasitica* 2220-01 applied at anthesis), treatment 7 (Figure 3). The range of spikes/pot for Go was 10~16 spikes compared to a control of 12, for Brandon there was 12~15 spikes compared to a control of 11 spikes, for Strongfield there was 10~12 spikes compared to a control of 11, and for Credence, there was 9~15 spikes compared to a control of 10. The highest increase in spike number of 44% with a single BCA treatment was observed in the wheat cultivar, Brandon, with treatment 7, *Sm*X_seed_, and the lowest reduction of 28% was observed in the durum cultivar, Credence, also with treatment 7, *Sm*X_seed_. Treatment 2, *Sm*_seed_, was effective across all four (common and durum wheat) cultivars, while *Sm*X_seed_, treatment 7, was more effective in the common wheat cultivars. For the durum wheat cultivars, treatments 5, *Sm*Pro _anth_ (*S. mycoparasitica* 2220-01 and fungicide applied at anthesis) and 4, *Sm*_anth_, were more effective than others when compared to the control.

#### 2.1.2. Spike Weight

**Figure 4:**
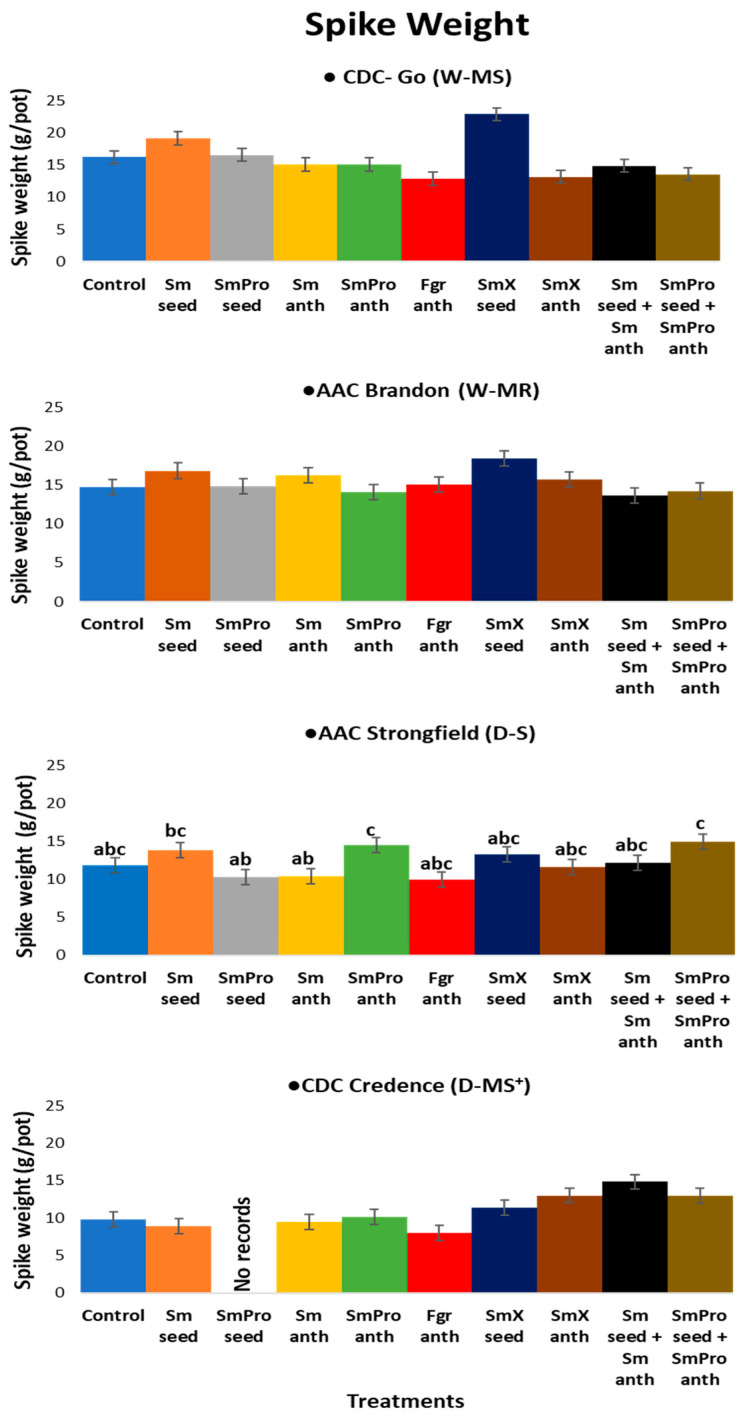
Effect of treatments on average spike weight/pot of four varieties, i.e., two common wheat species—CDC Go moderately susceptible (W-MS) and AAC Brandon, moderately resistant (W-MR) and two durum wheat species—AAC Strongfield susceptible (D-S) and CDC Credence, intermediately/moderately resistant (D-MS^+^/D-MR) cultivars. The data for each variety were statistically analyzed using One-way Analysis of Variance (ANOVA) with Duncan’s Multiple Range Test (DMRT) at *p* = 0.05. The means and standard deviations are represented by error bars. All plants in all treatments except for the control and *Fgr*_anth_ were inoculated with *Fusarium graminearum* within an 8 h window after the application of the BCA treatment. There were five plants per pot for each treatment in each cultivar. Each treatment for each cultivar had three replicates.

The average spike weight for each cultivar was Go (W-MS)—15.93 g/pot, Brandon (W-MR)—15.43g/pot, Strongfield (D-S)—12.26 g/pot, and Credence (D-MS^+^/D-MR)—9.82 g/pot. The highest spike weight for Go—22.85 g, and Brandon—18.48 g was observed with treatment 7, *Sm*X_seed_ (Figure 4). Strongfield had the highest spike weight with treatments 2, *Sm*_seed_ and 5, *Sm*Pro_anth_ (Figure 4). For Credence, *Sm*_seed_ and *Sm*_anth_ promoted the highest spike weight (Figure 4). The treatment of *Fusarium graminearum* at anthesis (*Fgr*_anth_) resulted in the lowest spike weight in all four cultivars. Among the four cultivars, Credence had the lowest spike weight/pot of 7.94 g, while Go had the highest single plant spike weight of 22.85 g/pot (Figure 4). The highest increase in spike weight of 52% with any single BCA treatment was observed in the intermediately/moderately resistant durum wheat cultivar, Credence, with treatment 9, *Sm*_seed_ + *Sm*_anth_ (*S. mycoparasitica* 2220-01 seed + *S. mycoparasitica* 222-01 at anthesis), and the most significant reduction of 21% was seen in wheat cultivar, Go, with treatment, *Fgr*_anth_. Treatments, *Sm*_seed_ and *Sm*X_anth_, were most effective in the common wheat cultivars, while treatments, SmPro_anth_ (5), *Sm*_seed_ + *Sm*_anth_ (9), and *Sm*Pro_seed_ + *Sm*Pro_anth_ *Sm*Pro_anth_ (*S. mycoparasitica* 2220-01 and fungicide applied both at the seed stage and anthesis) (10), were the most effective in durum wheat when compared to the control (Figure 4).

#### 2.1.3. Seed Yield

**Figure 5:**
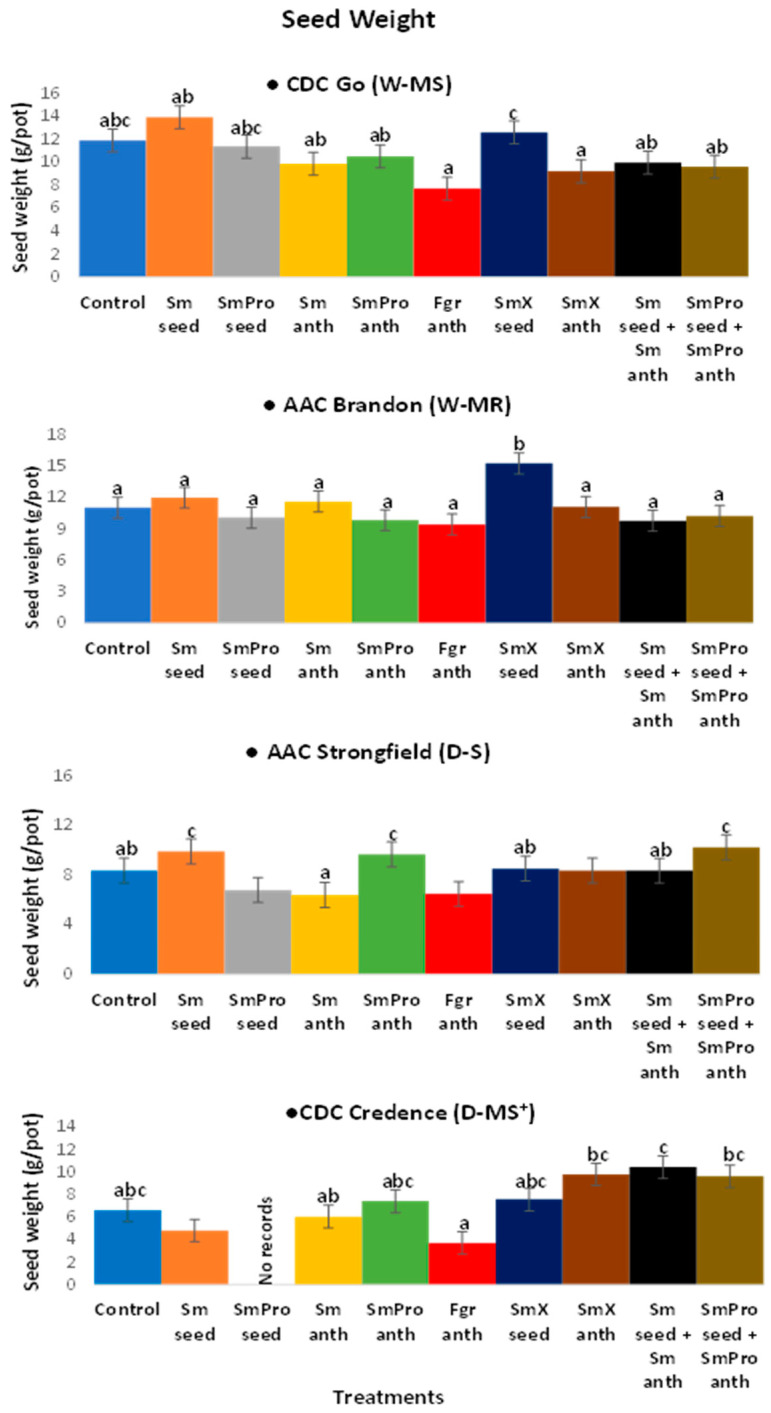
Effects of treatments on harvested seed weight or yield (g/pot) of two common wheat species—CDC Go moderately susceptible (W-MS) and AAC Brandon, moderately resistant (W-MR) and two durum wheat species—AAC Strongfield susceptible (D-S) and CDC Credence, intermediately/moderately resistant (D-MS^+^/D-MR) cultivars evaluated under greenhouse conditions. The data for each variety were statistically analyzed using One-way Analysis of Variance (ANOVA) with Duncan’s Multiple Range Test (DMRT) at *p* = 0.05. Means and standard deviations are represented by error bars. The same letters in each variety are not statistically different. The *p-*values for the varieties are CDC Go (0.04); AAC Brandon (0.01); AAC Strongfield (0.02); CDC Credence (0.01). All plants in all treatments except for Control and *Fgr*_anth_ were inoculated with *F. graminearum* within an 8 h window after the application of the BCA treatment. There were five plants per pot for each treatment in each cultivar. Each treatment for each cultivar had three replicates.

The average harvested seed weight or yield (g/pot) for each cultivar was Brandon—11.0 g/pot, Go—10.7g/pot, Strongfield—8.3 g/pot, and Credence—6.6 g/pot. For Go, the average harvested seed yield ranged from 7.7~13.9 g/pot compared to the control of 11.9 g/pot, while for Brandon, the range was 9.4~15.3 g/pot compared to the control of 11.0 g/pot. For the durum cultivars, the average harvested seed weight range for Strongfield was 6.4~10.2 g/pot compared to the control of 8.4 g/pot and for Credence, it was 4.8~10.4 g/pot compared to the control of 6.6 g/pot (Figure 5). The average seed yield of 13.9 g/pot for the cultivar, Go (W-MS) was associated with treatment 2-*Sm*_seed_, while the highest average seed yield of 10.2 g/pot for Strongfield (D-S^+^) was associated with treatment 10, *Sm*Pro_seed_ + *Sm*Pro_anth_ (Figure 5). For Brandon (W-MR), the highest average seed yield of 15.3 g/pot was associated with treatment 7, *Sm*X_seed_. For Credence (D-MS^+^/D-MR), treatment 2, *Sm*_seed_, and treatment 4, *Sm*_anth_, promoted the highest average seed yield of 10.4 g/pot. For all cultivars, the lowest seed yield and the highest FHB incidence were associated with the *Fusarium* artificial inoculation treatment 6 (Figure 5). The greatest reduction in average seed yield as a result of FHB damage ranged from 40~60%. The highest increase in harvested average seed yield in any cultivar with a single BCA treatment was 58% in Credence (D-MS^+^/D-MR).

#### 2.1.4. Vegetative Plant Biomass

**Figure 6:**
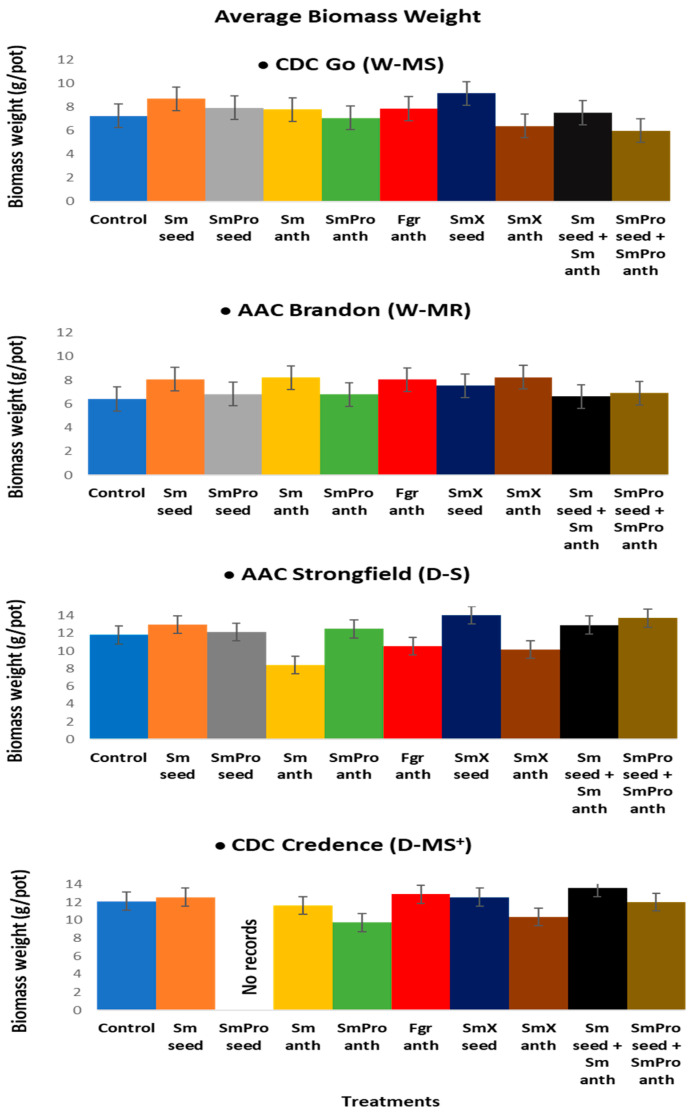
Effects of treatments on average biomass (g/pot) of four cultivars, i.e., two common wheat species—CDC Go moderately susceptible (W-MS) and AAC Brandon, moderately resistant (W-MR) and two durum wheat species—AAC Strongfield susceptible (D-S) and CDC Credence, intermediately/moderately resistant (D-MS^+^/D-MR) evaluated under greenhouse conditions. The data for each variety were statistically analyzed using One-way Analysis of Variance (ANOVA) with Duncan’s Multiple Range Test (DMRT) at *p* = 0.05. The means and standard deviations are represented by error bars. All plants in all treatments except for Control and *Fgr*_anth_ were inoculated with *Fusarium graminearum* within an 8 h window after the application of the BCA treatment. There were five plants per pot for each treatment in each cultivar. Each treatment for each cultivar had three replicates.

The average biomass (g/pot), which is the weight of the aerial (foliar) plant parts (leaves and stems without seeds) measured after harvest for each cultivar, was Go—10 g/pot, Brandon—7.4 g/pot, Strongfield—11.9 g/pot, and Credence—10.7 g/pot. The average biomass for Go ranged from 6.0~9.1 g/pot compared to Control of 7.3 g/pot, while for Brandon, the range was 6.8~8.2 g/pot compared to Control of 6.4 g/pot. For the durum cultivars, the average biomass range for Strongfield was 10.1~14.0 g/pot compared to Control of 11.8 g/pot and for Credence, it was 9.7~13.6 g/pot compared to Control of 12.1 g/pot, (Figure 6). Go (W-MS) and Strongfield (D-MS) had the highest average biomass (g/pot) with treatment 7, *Sm*X_seed_, of 9.15 g/pot and 14.0 g/pot, respectively. For Brandon (W-MR), the highest average biomass of 8.2 g/pot was recorded with *Sm*X_anth_, treatment 8. For Credence (D-MS^+^/D-MR), the highest average biomass of 13.6 g/pot was recorded with treatment 9, *Sm*_seed_ + *Sm*_anth_ (Figure 6). The lowest average biomass of 6.4 g/pot for Brandon was observed in the control group of plants. For Strongfield, *Sm*_anth_ treatment 2 resulted in the lowest biomass of 8.4 g/pot while in the cultivar Credence, the lowest average biomass of 9.7 g/pot was observed with treatment 5, *Sm*Pro_anth_ (Figure 6). Strongfield had the highest single average biomass, while Brandon had the lowest single average biomass. The highest increase in average biomass among the four cultivars for a single BCA treatment was 28% in Brandon, while the lowest reduction of 19.6% in average biomass with a single BCA treatment was seen in Credence.

#### 2.1.5. Plant Height

**Figure 7:**
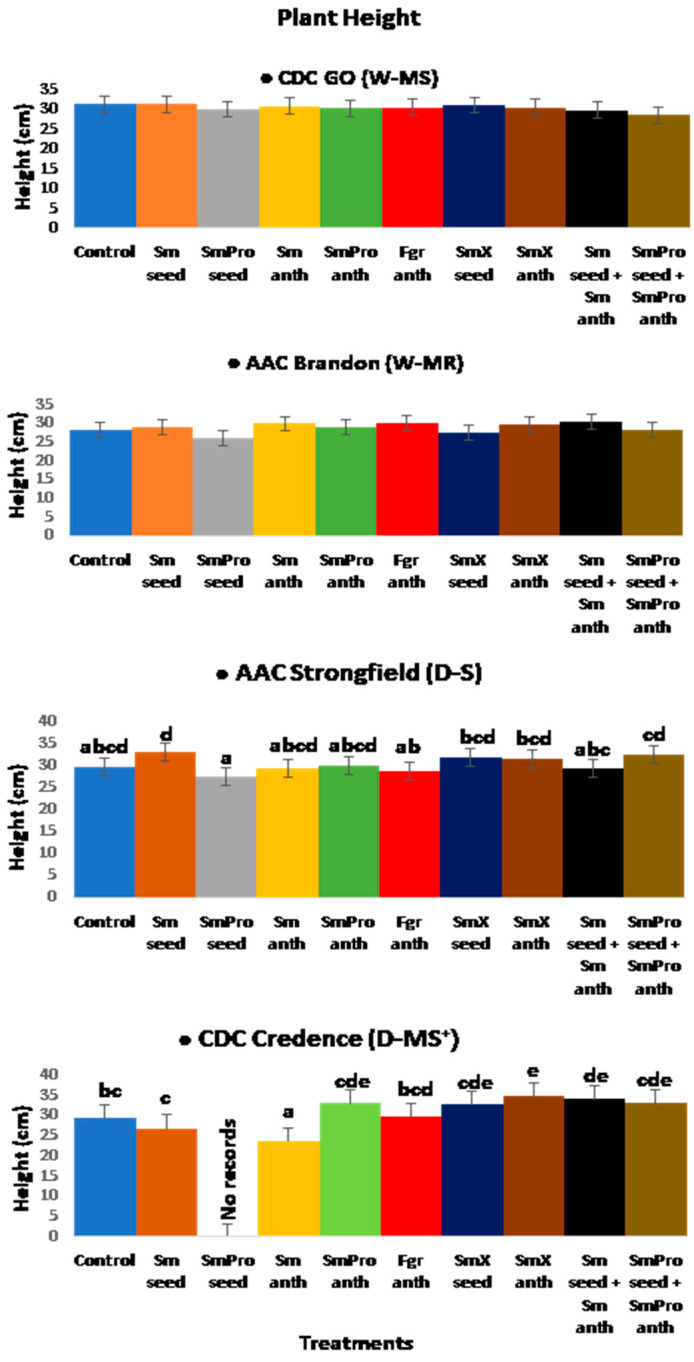
Effects of treatments on plant height (cm) of two common wheat species—CDC Go moderately susceptible (W-MS) and AAC Brandon, moderately resistant (W-MR) and two durum wheat species—AAC Strongfield susceptible (D-S) and CDC Credence, intermediately/moderately resistant (D-MS^+^/D-MR) cultivars evaluated under greenhouse conditions. The data for each variety were statistically analyzed using One-way Analysis of Variance (ANOVA) with Duncan’s Multiple Range Test (DMRT) at *p* = 0.05. Means and standard deviations for three replicates are represented by error bars. The same letters in each variety are not statistically different. The *p*-values for the varieties were as follows: AAC Strongfield (0.003): CDC Credence (0.0004). There were five plants per pot for each treatment in each cultivar. Each treatment for each cultivar had three replicates.

The average plant height (cm) for each cultivar was Go—30.5 cm, Brandon—28.4 cm, Strongfield 30.4 cm, and Credence 27.8 cm. For Go, plant height ranged from 28.6~31.2 cm compared to the control of 31.3 cm, while for Brandon, the range was 27.6~30.6 cm compared to the control of 28.2 cm. For the durum cultivars, plant height ranged from 27.5~33.1 cm for Strongfield compared to the control of 29.7 cm, and for Credence, it was 26.9~34.1 cm compared to the control of 29.3 cm (Figure 7). In Go, both the control and BCA seed-treated groups generated the tallest plants of 31 cm. The tallest plant of 30.6 cm for Brandon was observed with *Sm*_seed_ and *Sm*_anth_ treatments. For Strongfield, the tallest plants of 33.1 cm were observed with the *Sm*_seed_ treatment. The tallest single plant of 34.9 cm and the shortest single plant of 23.8 cm of all the treatments and cultivars were seen in Credence with treatment *Sm*X_anth_ (*S. mycoparasitica* SMCD 2220-01+ SMCD 2220-02(5)—a mixture of BCA beneficial strains applied at anthesis) and *Sm*_anth_, respectively (Figure 7). No significant increase in plant height was observed with the treatment *Sm*Pro_seed_ (*S. mycoparasitica* 2220-01+ fungicide applied to seeds), for the cultivars Go, Brandon, and Strongfield. For all five agronomic traits, no results were recorded for Credence with treatment 3, *Sm*Pro_seed_, as all the plants for that BCA treatment with chemical fungicide failed to grow to maturity.

Of the four varieties, Credence had some of the tallest plants, while Go had the highest overall average. The average plant height for Brandon was lower than that of the other cultivars. The highest increase in plant height of 19.1% with any single BCA treatment was observed in the intermediately/moderately resistant durum cultivar, Credence, with treatment *Sm*X_anth_, and the greatest reduction of 19% was also seen in Credence.

Overall, the ANOVA analyses of variance (Table 2) showed that variety and treatment had significant effects on all five traits of interest, and their interaction had a significant effect on all except for spike number. The result was consistent with the graphical results (Figure 3, Figure 4, Figure 5, Figure 6 and Figure 7) suggesting that each variety had a different response to the treatments. Indeed, when comparing the results based on the average values across all treatments, Go (W-MS) had the highest spike weight and spike number, Strongfield (D-S) had the highest biomass, Brandon had the highest seed yield, and Strongfield and Credence had the tallest plants. Of all four varieties, Credence (D-MS^+^/D-MR) had the lowest spike weight and seed yield, Strongfield (D-S) had the lowest spike number, and Brandon (D-MR) had the lowest biomass weight and plant height. For the treatment effects, *Sm*_seed_, *Sm*X_seed_, and *Sm*_seed_ + *Sm*_anth_ were the most effective of all treatments across all varieties in promoting a significant increase in spike weight, spike number, seed yield, plant biomass, and plant height compared to the Control. These three treatments appeared to be the most effective against FHB on plants grown under greenhouse conditions.

### 2.2. Fusarium Control in Susceptible vs. Resistant Cultivars

**Figure 8:**
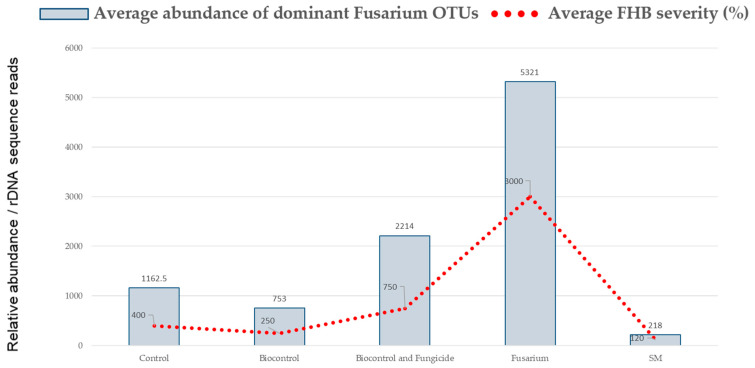
Relative abundance of the two dominant *Fusarium* OTU#2 and OTU#18 representing *F. graminearum* inoculant mixture of 3ADON SMCD2243 and SMCD2910–10B pathogenic strains, respectively. It is based on the number of rDNA sequence reads in (**A**) all tested CDC Go, CDC Credence, AAC Brandon, AAC Strongfield cultivars (blue bar) associated with FHB severity (%); (**B**) wheat (Go and Brandon) versus durum (Strongfield and Credence) cultivars: and (**C**) susceptible (Go and Strongfield) versus resistant (Brandon and Credence) cultivars throughout treatments. The treatments used were *Control*—no biocontrol agents (BCAs) or any other treatment; Biocontrol—*Sm*_seed_, BCA (*Sphaerodes mycoparasitica*)—applied to the seeds, *Sm*_anth_ *− S. mycoparasitica* applied at anthesis, *Sm*_seed_ + *Sm*_anth_ − *S. mycoparasitica* seed + *S. mycoparasitica* at anthesis; *Biocontrol and Fungicide*—*Sm*Pro_seed_ *− S. mycoparasitica* + Fungicide applied to seeds, *Sm*Pro_anth_ *− S. mycoparasitica* + Fungicide at anthesis, and *Sm*Pro_seed_ + *Sm*Pro_anth_ *− S. mycoparasitica* + Fungicide applied to seeds and *S. mycoparasitica* + Fungicide at anthesis; *Fusarium* (*F. graminearum* inoculant mixture of 3ADON SMCD2243 and SMCD2910–10B pathogenic strains) − *Fgr*_anth-_ a mixture of *Fusarium* applied at anthesis; *SM* (*Sphaerodes* mixture of beneficial strains)—*Sm*X _seed_ *− S. mycoparasitica* SMCD 2220-01 strain + *S. mycoparasitica* SMCD 2220-02(5) applied to seeds, and *Sm*X_anth_ ‒ *S. mycoparasitica* SMCD 2220-01 strain + *S. mycoparasitica* SMCD 2220-02(5) applied at anthesis. There were five plants per pot for each treatment in each cultivar. Each treatment for each cultivar had three replicates.

The Illumina sequencing results depicted two dominant *F. graminearum * Operational Taxonomic unit (OTU), OTU 2 and OTU 18, with the blasted ITS rDNA sequences, a 100% match with *F. graminearum* SMCD2243 clade F7 (GenBank under accession number HQ333185) and SMCD2910–10B clade F10 (GenBank under accession number HQ333188) inoculant strains, respectively. In Figure 8A, the reduction in the average *F. graminearum *abundance and FHB severity are presented showing differences between the effect of BCA—single beneficial strain (*Sm.* SMCD 2220-01) treatment versus the effect of the *Sm*X- *Sphaerodes *mixture of two beneficial strains (*Sm.* SMCD 2220-01 + *Sm.* SMCD 2220-02(5)). The *Sm*X treatment resulted in the lowest abundance of the dominant *F. graminearum* OTU inoculants even when compared to the control. The *Sm * and *Sm*X reduced the endogenous *Fusarium* population in naturally contaminated, non-treated seeds. The *Fusarium* (*Fgr*) artificial inoculant with a consortium or mixture of pathogenic strains had the highest abundance of the dominant *F. graminearum* OTU inoculants compared to control and all other treatments, as expected. However, the abundance of the dominant *F. graminearum* OUT in *Sm*Pro_anth_ (treatment 5) was higher than that of the control. The presence of synthetic fungicides coincide with lowering BCA efficacy against FHB disease. Overall, a higher abundance of the dominant *F. graminearum* OTU (FUS OTU2—3ADON SMCD2243 and FUS OTU18—SMCD2910–10B) inoculant strains was observed in the common wheat hosts compared to the durum wheat host (Figure 8B). However, an equal and relatively low *Fusarium* infection level was measured in common wheat and durum wheat non-treated control seeds and *S. mycoparasitica*-treated seeds (*Sm*_seed_). Biocontrol application on seeds based on a single *Sphaerodes* strain and a mixture of *S. mycoparasitica* beneficial strains (*Sm*X_seed_) was demonstrated to be the most efficient measure compared to *Fusarium* application followed by the Biocontrol and Fungicide (*Sm*Pro_anth_) treatment. Indeed, in both common wheat and durum wheat, an increase in *F. graminearum* OTUs was observed for BCA combination with Prosaro synthetic fungicide (*Sm*Pro). Further, in durum (tetraploid) wheat, all treatments resulted in a lower abundance of the dominant *F. graminearum* OTUs compared to non-treated control, which was the opposite in common (hexaploid) wheat. Overall, it seemed that the BCA treatments were effective in reducing *Fusarium* abundance when compared to both positive and negative controls, particularly in common wheat.

Further, the susceptible cultivars treated with *Sm* and *Sm*X treatments were associated with a lower abundance of the dominant *F. graminearum* OTUs compared to the untreated control (Figure 8C). While the *Sm*Pro treatment was highly effective in reducing *Fgr* OTUs in susceptible cultivars (common wheat and durum wheat), the opposite trend was recorded in all tested resistant (common wheat and durum wheat) cultivars.

## 3. Discussion

In wheat and other small cereals such as barley and oats, Fusarium head blight (FHB) affects the main agronomic characteristics linked to disturbances in grain development resulting in yield loss. To combat this loss, effective disease control has been contemplated. Over the years, research into various FHB management strategies has shown that the suppression of FHB in wheat positively correlates to an increase in grain production or caryopsis yield [19,20,25]. This study was carried out to investigate and compare the effects of a fungal endophytic biocontrol agent (BCA), *Sphaerodes mycoparasitica*, in suppressing FHB effects in five important agronomic traits that are linked to grain yield in elite common wheat and durum wheat cultivars that are commercially available in Canada. Although both common wheat and durum wheat are susceptible to *Fusarium* infection, it is widely accepted that tetraploid durum wheat is more susceptible than hexaploid common wheat due to a lack of inherent FHB resistance genes. This study aimed to figure out whether beneficial BCA can, in synergy with the host genotype, increase the FHB control effectiveness.

### 3.1. BCA Effect on Agronomic Traits

The BCA, *S. mycoparasitica*, a biotrophic mycoparasite, suppressed *Fusarium graminearum* and improved five different agronomic traits that are known to influence grain yield in wheat. These findings are consistent with previous work done on the Fusarium-specific mycoparasite, *S. mycoparasitica* [21,23]. This mycoparasite engages in biotrophic mycoparasitism by directly penetrating living *Fusaria* fungal cells with its haustoria [26]. This subsequently leads to the absorption and depletion of *Fusarium* nutrients and eventually death [21,23]. This action by the mycoparasite prevents the spread of *Fusarium* infection to surrounding plant cells. The findings of this study revealed that *S. mycoparasitica* improved spike weight, spike number, seed yield, biomass weight, and plant height which are important factors for grain yield in the two common wheat cultivars, moderately susceptible Go and moderately resistant Brandon, and the two durum wheat cultivars susceptible Strongfield and intermediately/moderately resistant Credence. Of the eight *S. mycoparasitica* inoculant formulations used in this study, *Sm*X_seed_—a mixture of two *S. mycoparasitica* strains applied to seeds, *Sm*_seed_ + *Sm*_anth_ −— *S. mycoparasitica* applied to seeds and then at the flowering stage, and *Sm*_seed_—*S. mycoparasitica* applied to the seeds were most effective in improving the five agronomic traits in all four cultivars.

For the agronomic trait spike weight, *Sm*X_seed_ was the most effective treatment in common wheat and *Sm*_seed_ + *Sm*_anth_ was the most effective in durum wheat. The greatest overall improvement in this agronomic trait was seen in the durum wheat cultivar, Credence, when compared to the control treatment. Even though the highest average of all four cultivars was recorded for the moderately susceptible wheat cultivar, Go, its average percentage increase for this trait was lower than that of both durum wheat cultivars. Moreover, most of the *S. mycoparasitica* treatments consistently improved the spike weight in the durum wheat cultivar, Credence, compared to Go and other cultivars. This finding is not comparative to any other research because at this time there are no similar studies reported in the literature.

Recent results suggest that spike characteristics are of great value for marker-assisted selection (MAS) in breeding programmes [27] and will accelerate the understanding of the genetic relationships among spike-related traits and the environment [28,29]. Thus, targeting reproductive and spike traits for the improvement in grain yields in wheat shows agronomic promise. However, spike production as a desirable agronomic trait, is usually reduced subsequent to *Fusarium* infection. In this study, Brandon was the most improved cultivar with a 44% increase in spike number compared to the control. The average spike number and spike weight were higher for Go than for Brandon. The study results show that the *Sm*X_seed_ treatment was particularly efficient in both common wheat species, Go and Brandon. In terms of the most effective BCA treatments, the maximum efficacy was obtained by *Sm*X_seed_ in common wheat and *Sm*Pro_anth_ in durum wheat. Finally, the BCA was more effective in improving the spike production in common wheat than in durum wheat.

The treatment, *Fgr*_anth_, a mixture of *Fusarium graminearum* 3ADON strains applied at anthesis, resulted in the greatest percentage reduction in spike weight in all cultivars except for Brandon, where the *Fgr*_anth_ treatment slightly improved the spike weight. At first glance, this was a bit surprising but somewhat counterintuitive, considering that Brandon is the most resistant cultivar in this study and one of the more resistant cultivars in Canada at the time of this study. It is possible that Brandon’s resistance mechanisms may have allowed the infected plant to thrive and grow in the presence of the pathogen, *F. graminearum*. However, the increase was a minimal 2% when compared to the control and other *S. mycoparasitica*-formulated products. This improvement in spike weight in the presence of *F. graminearum* was not only limited to this trait with this cultivar Brandon, but four of the five agronomic traits were not adversely affected by the pathogen, which is in stark contrast to what was seen with this treatment in the cultivars, Go and Credence. The BCA treatments were more effective in improving this agronomic trait in durum wheat cultivars compared to common wheat cultivars.

Harvested seed or crop yield is also another agronomic trait that is highly affected by *Fusarium* infection. Upon *Fusarium* infection and the colonization of tissues, the pathogen depletes the host’s resources, resulting in very light, hollow, shrivelled, diseased seeds, reduced grain yield, and downgraded grains. FHB and accumulated mycotoxins affect grain nutrient quality, safety, and security, and minimize the bioeconomy in farms. This trait was mostly improved by *Sm*_seed_ + *Sm*_anth_ in durum wheat and *Sm*X_seed_ in common wheat. The most improved cultivar by 58% for this trait was intermediately/moderately resistant Credence when compared to the control. As with spike weight, the hexaploid wheat cultivars had on average a higher seed yield but their percentage increase, when compared to their respective controls, was lower than that of the durum cultivars, Credence and Strongfield. For this trait, some plants undergoing *Sm*Pro_seed_ treatment showed a detectable sensitivity to the synthetic fungicide used in combination with BCA. The treatment, *Fgr_anth_*, significantly reduced this trait in all four cultivars ranging from 15–44% indicating a possibility of the suppressive chemical environment for BCA and/or reduced FHB resistance in the tested crop varieties. For harvested seed yield, *S. mycoparasitica* was more effective in tetraploid durum wheat.

The plant biomass was improved by the BCA treatments of *Sm*X_seed and_ *Sm*X_anth_ in common wheat, and *Sm*X_seed_ and *Sm*X_seed_ + *Sm*X_anth_ in durum wheat. The greatest improvement for this trait was seen with the cultivar, Brandon, which had a net increase after all treatments when compared to the control. The durum cultivars had a higher average plant biomass, but when compared to their respective controls, the percentage improvement for this trait was lower than that of Brandon. For this trait, *S. mycoparasitica* formulations were more effective in common wheat cultivars compared to durum wheat cultivars.

### 3.2. Common Wheat versus Durum Wheat

In wheat, research has shown that shorter plants have been associated with increased grain yields. There is also considerable research validating the effects of the introgression of dwarfing genes into crops to improve grain yields [28]. However, in the case of FHB resistance, shorter plants are usually in closer proximity to the soil surface, which most times is the source of the *Fusarium* inoculum. Therefore, taller wheat plants are usually considered to have a form of passive resistance to FHB infection and disease progression. In this study, plant height was the least improved of all the agronomic traits. The most effective *S. mycoparasitica* treatment in common wheat was *Sm*_seed_ + *Sm*_anth_, while *Sm*X_anth_ was more effective in durum wheat. The most improved cultivar with a 19% increase was Credence followed by Strongfield. These durum wheat cultivars had the tallest plants, the highest plant height averages, and the greatest percentage increase when compared to their respective controls and the common wheat cultivars. The highest increase in Strongfield was 11%. There was no net increase in Go and the highest increase in Brandon was 9% with treatment *Sm*_seed_ + *Sm*_anth_. Regarding Go, there was some yield reduction observed in plants treated with BCA (Prosaro) (*Sm*Pro_seed_ + *Sm*Pro_anth_) fungicide. This corroborates recent Canadian field study results that showed that some wheat cultivars yielded significantly less in the fungicide-treated compared with the untreated plots [29,30]. In addition, Caldwell et al. [31] reported that a single fungicide application against FHB was not sufficient to achieve a high wheat yield with good seed quality, which qualified BCA as a more ecologically friendly solution to control FHB compared to chemical fungicides.

In the durum cultivars, the treatment *Sm*Pro_seed_ + *Sm*Pro_anth_ was one of the more consistent BCA formulations that was associated with significant improvements in all five traits for the cultivar, Strongfield, and a minimal reduction in two agronomic traits in the cultivar, Credence. This was also in stark contrast with other BCA treatments such as the *S. mycoparasitica* only applied to seeds (*Sm*_seed_) or *Sm*X_seed_ treatment that improved all agronomic traits in Go. For plant height, durum wheat was again the more improved species by *S. mycoparasitica* compared to common wheat. It has been established in numerous studies that durum cultivars are more susceptible to *Fusarium* infection and subsequent mycotoxin formation than common wheat and other small-grain cultivars, and thus far less effort has been made to improve the resistance in durum wheat. The findings of numerous studies have also revealed that Biocontrol agents (BCAs) are usually more effective in FHB tolerant or resistant wheat and other cereal crops [32,33,34]. Therefore, it would be intuitive to hypothesize that *S. mycoparasitica* would be more effective in improving agronomic traits such as spike number, spike weight, harvested seed weight/seed yield, biomass, and plant height which are usually affected by *Fusarium* infection in the FHB tolerant/resistant wheat cultivars. However, this was not always clear from the results of this research. While the moderately susceptible wheat cultivar, Go, benefited significantly from the different BCA treatments and showed considerable improvement in the observed agronomic traits, the wheat cultivar, Brandon, one of Canada’s most resistant cultivars, was one of the least improved of all cultivars and showed some of the lowest improvement in different agronomic categories. Vujanovic et al. (2021) reported that wheat kernel yield may be influenced by the plant symbiotic mycobiome, particularly the coexistence of the endophytic mycoparasite, *S mycoparasitica*, with endophytic plant growth-promoting fungi and yeasts [20].

In general, the BCA (*S. mycoparasitica*) treatments had a more significant effect on durum cultivars compared to common wheat cultivars when their respective (non-treated and only *Fgr_anth_*-treated seeds) controls were used as the base of comparison. For the agronomic trait spike weight, the highest percentage increase of 52% with any treatment and in all cultivars was observed in the intermediately/moderately resistant Credence with the treatment *Sm*_seed_ + *Sm*_anth_, followed by Go with an increase of 41% with the treatment, *Sm*X_seed_. The cultivar, Credence, also had the highest percentage increase of 58% in seed yield with the treatment, *Sm*_seed_ + *Sm*_anth_, followed by Brandon with an increase of 38.2% with the treatment, *Sm*X_seed_. The cultivar, Credence also had the highest percentage increase in plant height with the treatment, *Sm*X_anth_, but it also had the greatest reduction in the same agronomic trait with the treatment, *Sm*_seed_. The *Sphaerodes mycoparasitica*-only treatment and others were also more effective in improving the agronomic traits in durum wheat than in common wheat. A possible explanation for this is that common wheat has an extensive history of FHB infection and so has a more primed resistance. In addition, this common wheat’s hexaploid genome is larger, and more efforts have been made to improve the resistance in this species. Conversely, durum has a smaller genome, a shorter history with FHB, and subsequently a less primed resistance. This inherent shortcoming leaves room for improvement that can be filled by a protocooperative, balanced biotrophic mycoparasite such as *S. mycoparasitica*. This BCA is a polyphagous mycoparasite, that specifically parasitizes different species of *Fusarium* including *F. graminearum* [21], while common wheat and durum wheat are the perfect hosts for *Fusarium* pathogens, the main carbon source of this mycoparasite. Therefore, as *Fusarium* increases in the host, especially durum, where inherent FHB resistance is highly limited, the reduction in pathogenic *Fusarium* DNA [35,36,37,38] and degradation of mycotoxins [18] is likely to increase, resulting in a greater improvement in durum cultivars. Also, this BCA as an endogenous mycoparasite not only destroys *Fusarium* but also possibly boosts the plant’s defences [20] by inducing the plant’s immune system to regulate or switch on different resistance responses. In this way, it acts as a protector, especially from the seed stage.

### 3.3. BCA Effect on Fusarium graminearum in Seeds

As the primary pathogenic agent in the aetiology of the FHB disease, *Fusarium graminearum* infection occurs predominantly in the developing spikes in wheat and other small cereals during the flowering stage when the anthers are extruded and serve as the perfect nutrient source for the pathogen. According to Mesterházy [39], it is imperative that wheat cultivars can generate spikes fortified with an effective defence mechanism to be at least able to mount a Type I resistance to initial infection or Type II resistance to fungal spread in the wheat head subsequent to infection. At present, to our knowledge, there is little to no research comparing the effects of fungal biocontrols on FHB in both common wheat and durum wheat. Therefore, this study investigated the BCA effects on agronomic traits, namely spike number, spike weight, seed weight, plant biomass, and seed weight under high *Fusarium* pressure using a mixture of aggressive 3ADON strains for artificial inoculation of wheat.

In this study, treatments *Sm*_seed_ and *Sm*X_seed_ seemed to have a better effect in reducing the dominant *F. graminearum* OTUs in durum wheat and susceptible cultivars compared to common wheat and resistant cultivars, respectively. It seemed that synthetic fungicide addition to BCA treatment has less effect on the dominant FUS OTUs compared to BCA applied alone on common wheat, while no negative effect was registered in durum cultivars under greenhouse conditions. Further analyses should evaluate the effect of synthetic chemical fungicide treatment alone and in combination with BCA under field settings. Overall, *Fusarium* analyses associated with greenhouse-grown plants in common wheat and durum wheat cultivars indicated a significant effect of combined cultivars and BCA application treatments, as indicated by the ANOVA analysis (Table 2) based on FHB incidence (Figure 8A) and *Fusarium* abundance (Figure 8B,C) parameters.

## 4. Materials and Methods

### 4.1. Cultivars and Treatments

Four wheat cultivars, two common wheat species, CDC Go (moderately susceptible, W-MS) and AAC Brandon (moderately resistant, W-MR), and two durum wheat species, AAC Strongfield (moderately susceptible, D-MS) and CDC Credence (intermediary/moderately resistant, D-MS^+^/D-MR), were used in this study. The CDC Credence D-MS^+^/D-MR abbreviation describes a susceptible plus (D-MS^+^) durum wheat cultivar, which is a term recently revised to define an intermediately or moderately resistant (D-MR) durum wheat cultivar according to the Saskatchewan Ministry of Agriculture guide, entitled “Varieties of Grain Crops”; see the link: https://saskseed.ca/wp-content/uploads/2020/12/Seed-Guide-Varieties-Section-2021.pdf. (accessed on 15 Novembre 2022.) For each variety grown in the greenhouse experiment, 10 treatments were used in the evaluation of major agronomic traits: spike number, spike weight, seed weight, biomass, and plant height. [The acronym CDC stands for Crop Development Centre, while AAC stands for Agriculture and Agri-Food Canada]. Seeds for each cultivar were provided by the Crop Development Centre, The University of Saskatchewan.

### 4.2. Greenhouse Trials and Sampling Methods

All potted common wheat and durum wheat plants were grown in an AgBio College greenhouse facility (University of Saskatchewan) rooms separated from those harbouring other plant hosts. Seeds (provided by Dr. Hucl of CDC, University of Saskatchewan) were surface disinfected in 75% ethanol for 10 s, rinsed with sterile distilled water for 10 s, submerged for 3 min in 5% sodium hypochlorite (Javex^®^ 12 Bleach), and then rinsed five times with sterile distilled water. There were 10 treatments in total as described in Table 1. For each treatment, five seeds were placed in one 4 L plastic pot containing 400 g (dry weight) of autoclaved field-capacity Sunshine mix 4 (SunGro Horticulture Canada Ltd., Vancouver, BC, Canada) consisting of three replicates. For treatments in which the BCA was applied at the seed stage, wheat seeds of each cultivar were surface-sterilized and coated with 48 h grown *Sphaerodes mycoparasitica* SMCD 2220-01 or a combination of SMCD 2220-01 and SMCD 2220-02(5) for treatment 7, *Sm*X_seed_ {SMCD—Saskatchewan Microbial Collection and Database, Saskatoon, Canada] [21]. The seeds were inoculated with the *S. mycoparasitica*—BCA and/or *F. graminearum*—3ADON pathogen, then covered with a ~4 cm layer of sterilized Sunshine mix 4 (SunGro Horticulture Canada Ltd., Vancouver, BC, Canada). In this experiment, the pots were arranged in a randomized complete block design (RCBD) in the greenhouse to mimic the setting in concurrent field trials. The locations of blocks of pots and/or individual pots were changed weekly to account for any variation in the greenhouse conditions. Day and nighttime temperatures over the summer varied from 20 to 36 °C and 16 to 20 °C, respectively. Relative humidity during the afternoon and nighttime varied from 50–90%. On sunny days, plants were exposed to the natural sunlight. On cloudy or winter days with reduced daylight photoperiodic conditions, 1000-watt high-pressure sodium light bulbs supplemented sunlight. These bulbs were suspended from the ceiling roughly 2 m above the plants. Standard-watered plants were kept at about 90–95% water-holding capacity (max. 100% only at the time of watering) [19] with 1300 ppm NPK (20-20-20) supplementation at 14-day intervals [24]. A typical application rate of *S. mycoparasitica* to *seeds* as a liquid suspension was 8 g per litre of water as the optimized suspension mix and 10 mL of that suspension (1 to 2 × 10^6^ CFU—Colony Forming Unit) was used to inoculate a kilogram of seeds. This pertains to all treatments in which the BCA was applied to seeds. For treatments in which the BCA was applied at the flowering stage, a typical foliar application range was 8–10 mL BCA + 60 mL of water per greenhouse experiment (Figure 1) due to the differences in crop size and maturity stage. The highest concentration of Fusarium graminearum used in this study was 1–2 × 10^4^ CFU/mL, as a mixture of *F. graminearum* 3ADON chemotype strain SMCD2243 and the *F. graminearum* 3ADON strain SMCD2910–10B, was applied to all treatments 2–9 at the flowering stage during an 8 h window after BCA application. The dosage of prothioconazole + tebuconazole (Prosaro^®^ 250 EC, Bayer) chemical fungicide [31] used in this study was 50% of the recommended concentration for the Raxil PRO application as suggested by the manufacturer, Bayer. A FLO-Master 1998TL Premium Home & Garden Sprayer (Lowell Manufacturing Co., Lowell, MI) sprayer was used to apply inoculants on healthy spikes. After spraying, the spikes were covered with transparent plastic bags for 12 h to ensure that each spike was fully contaminated by the inoculant. Fusarium head blight (FHB) was assessed according to the scale of *Fusarium* disease symptoms as shown in Figure 2. Harvested plants and seeds were also measured, weighed, and evaluated for five traits. Each treatment for each cultivar had 5 plants per pot with 3 replications in the greenhouse experiments.

### 4.3. Assessment of Fusarium graminearum in Mature Harvested Seeds

The effect of all treatments (Table 1) on the relative abundance of *Fusarium* 3ADON’s operational taxonomic units (OTUs) from an inoculant (*v*/*v* 50:50) mixture of SMCD2243 and SMCD2910–10B was assessed in common wheat and durum wheat seeds by using the previously reported Illumina MiSeq research approach [19,40]. The total genomic DNA was extracted from seed material using a plant DNA extraction kit (Qiagen Inc., Montréal, QC, Canada). Seed samples (~100 mg) were disrupted using the Precellys 24 Tissue Homogenizer (Bertin Instruments, Montigny-le-Bretonneux, France). DNA extractions were conducted following the manufacturer’s protocols. The DNA yield was quantified using the Qubit DNA HS Assay Kit (Thermo Fisher Scientific, Waltham, MA, USA) and DNA electrophoresis in 1% agarose gel stained with the SYBRTM safe DNA gel stain (Invitrogen, Waltham, MA, USA). DNA samples from replicates of the same treatment were pooled together to an equal DNA (3 × 100 ng) ratio. A total of 20 DNA samples, 10 per crop (wheat and durum) and 10 per genotype resistant type (susceptible and resistant) were submitted for high-throughput sequencing to the Génome Québec Innovation Centre, McGill University using Illumina MiSeq technology. The PCRs were conducted using the primers ITS1F (5′-CTTGGTCATTTAGAGGAAGTAA-3′) and ITS4 (5′-TCCTCCGCTTATTGATATGC-3′), which amplify the ITS fungal gene [41]. Sample libraries were prepared according to the MiSeq reagent kit preparation guide (Illumina, San Diego, CA, USA) and the sequencing protocol from de la Cuesta-Zuluaga and Escobar [42]. Statistical analyses were performed to assess dominant *Fusarium graminearum* OTUs in grain samples originating from the inoculant mixture of 3ADON (SMCD2243 and SMCD2910–10B) pathogenic strains. The ITS sequences were previously obtained from the pure culture of the two strains used in the composed inoculant. ITS sequences derived from extracted DNA from common wheat and durum wheat seeds using high-throughput Illumina technology were analyzed using Mothur version 1.34.3 [27]. The standard operating procedure included the generation of contigs from the combination of forward and reverse reads and the removal of sequence errors and chimeras [43]. Taxonomic classification was performed with a naïve Bayesian classifier using the SILVA database. Reads displaying at least 97% identity were clustered into Operational Taxonomic Units (OTUs).

### 4.4. Statistical Analysis

In the greenhouse, plant agronomic traits including spike weight, spike number, biomass weight, or harvested plant weight, seed weight, and plant height were evaluated after harvesting. Data collection and analyses followed Canadian crop studies on the control of FHB and its effect on wheat phenotypic characteristics as detailed in Caldwell et al. (2017) [40]. Analysis of variance (ANOVA) was performed using the PROC MIXED model [44] in the Statistical Analysis System (SAS version 9.3,) package [45]. The data (g/pot) or (cm/plant) variety were statistically analyzed using ANOVA with Duncan’s Multiple Range Test (DMRT) at a *p*-value of 0.05. Means and standard deviations for 3 replicates are represented by bars and error bars. Each plant trait including the seed weight, the harvested plant weight, the plant height, the spike weight, and the number of spikes are graphically presented. In addition, One-way ANOVA was conducted for the effect of treatment on each trait (Table 2). Even though an RCBD was used, the effect of any blocking factor was ruled out due to the weekly rotation of the blocks. The primary purpose of the experiment in the greenhouse was to determine how the BCA influenced trait development.

## 5. Conclusions

This study’s results based on the average values across all treatments depict CDC Go (W-MS) as having the highest spike weight and spike number, AAC Strongfield (D-S) as having the highest biomass, AAC Brandon as having the highest seed yield and AAC Strongfield, and CDC Credence as having the tallest plants. A *substantial variation* in the plant traits response across cultivars has been previously interpreted as an interplay between wheat cultivars, the competitiveness of endophytic and mycoparasitic symbionts, plant pathogens, biocontrol, soil, and climate change [19,20,46,47]. The BCA treatments that were most effective in improving all five agronomic traits were *Sm*X_seed_, *Sm*_seed_ + *Sm*_anth_, and *Sm*_seed_. The treatment, *Fgr*_anth_, was consistent in suppressing development in all five agronomic traits across all cultivars. The agronomic traits that were most improved by *S. mycoparasitica* were spike weight, spike number, seed weight, and ultimately yield. Coincidentally, these are the traits that are mostly affected by *Fusarium* infection and a reduction in these usually leads to major losses in total wheat production. Therefore, the result of this study is quite promising in that efforts can be made to improve these traits to provide greater control of FHB and crop resistance against *Fusarium* infection and spread.

## Figures and Tables

**Table 1:** List of treatments.

Type	Treatment	*Sphaerodes mycoparasitica* (*Sm*) (2220-01)		Fungicide(F) (Prosaro)		*F. graminearium* (F.gr) 3ADON Mixture of Strains		Additional Notes
Treatment Number & Name		Applied at Seed	Applied at Anthesis (anth)	Applied at Seed	Applied at Anthesis (anth)	Applied at Seed	Applied AT Anthesis (anth)	
1.	Control	-	-	-	-	-	-	No biocontrol agents (BCAs) or any other treatment
2.	*Sm*seed	Yes	-	-	-	-	Yes	BCA (*S. mycoparasitica* 2220-01) applied to seeds only
3.	*Sm*Pro seed	Yes	-	Yes	-	-	Yes	*S. mycoparasitica* 2220-01 + Fungicide applied to seeds (1/2 of the effective dose of fungicide; Prosaro was used)
4.	*Sm* anth	-	Yes	-	-	-	Yes	*S. mycoparasitica* 2220-01 applied at anthesis
5.	*Sm*Pro anth	-	Yes	-	Yes	-	Yes	*S. mycoparasitica* 2220-01+ Fungicide applied at anthesis
6.	*Fg* anth	-	-	-	-	-	Yes	*F. graminearum* 3ADON mixture of strains applied at anthesis
7.	*Sm*X seed	Yes	-	-	-	-	Yes	*S. mycoparasitica* SMCD 2220-01+ SMCD 2220-02(5) mixture of BCA beneficial strains, applied to seeds
8.	*Sm*X anth	-	Yes	-	-	-	Yes	*S. mycoparasitica* SMCD 2220-01 + SMCD 2220-02(5) applied at anthesis
9.	*Sm* seed + *Sm* anth	Yes	Yes	-	-	-	Yes	*S. mycoparasitica* 2220-01 applied to seeds + *S.mycoparasitica* 2220-01 applied at anthesis
10.	*Sm*Proseed+ *Sm*Pro anth	Yes	Yes	Yes	Yes	-	Yes	*S. mycoparasitica* 2220-01+ Fungicide applied to seeds and *S. mycoparasitica* 2220-01+ Fungicide applied at anthesis.

**Table 2:** Analysis of variance for 5 traits of interest in treatments tested on common wheat durum wheat using ANOVA.

		F Value				
Factor	DF	Biomass	Spikew	Spiken	Seedy	Height
Var	3	77.31 ***	27.09 ***	5.16 **	27.39 ***	5.59 **
Trt	9	2.26 *	3.85 **	2.17 *	5.07 ***	3.66 ***
Var × trt	26	1.56 *	2.00 *	1.44 ns	2.74 ***	3.97 ***

Note: var, variety; trt, treatment; biomass, biomass weight (g); spikew—spike weight (g); spiken—spike number; seedy—seed yield (g); height—plant height (cm); *, significant at 0.05; **, significant at 0.01; ***, significant at 0.001; ns, not significant.

## Data Availability

There are no data supplied other than what are represented in the Tables and Figures.

## References

[1] FAOSTAT (2021). World Food and Agriculture–Statistical Yearbook 2021.

[2] Crespo-Herrera L.A., Crossa J., Huerta-Espino J., Vargas M., Mondal S., Velu G., Payne T.S., Braun H., Singh R.P. (2018). Genetic Gains for Grain Yield in Cimmyt’s Semi-Arid Wheat Yield Trials Grown in Suboptimal Environments. Crop Sci..

[3] Sharma R.C., Crossa J., Velu G., Huerta-Espino J., Vargas M., Payne T.S., Singh R.P. (2012). Genetic Gains for Grain Yield in CIMMYT Spring Bread Wheat across International Environments. Crop Sci..

[4] United Nations (2019). World Population Prospects. Highlights.

[5] STATCAN (2023). Production of Principal Field Crops-Wheat 2023.

[6] Powell A.J., Vujanovic V. (2021). Evolution of Fusarium Head Blight Management in Wheat: Scientific Perspectives on Biological Control Agents and Crop Genotypes Protocooperation. Appl. Sci..

[7] Borlaug O.E. (1968). Wheat Breeding and Its Impact on World Food Supply. Ausl. Acad. Sci. Canberra.

[8] Hilton A.J., Jenkinson T., Hollins W., Parry D.W. (1999). Relationship between Cultivar Height and Severity of Fusarium Ear Blight in Wheat. Plant Pathol..

[9] Lahlali R., Kumar S., Wang L., Forseille L., Sylvain N., Korbas M., Muir D., Swerhone G., Lawrence J.R., Fobert P.R. (2016). Cell Wall Biomolecular Composition Plays a Potential Role in the Host Type II Resistance to Fusarium Head Blight in Wheat. Front. Microbiol..

[10] Mills K., Salgado J.D., Pierce P.A. (2016). Fusarium Head Blight or Head Scab of Wheat, Barley and other Small Grain Crops.

[11] Hucl P., Briggs C., Graf R., Chibbar R.N. (2015). Genetic Gains in Agronomic and Selected End-Use Quality Traits over a Century of Plant Breeding of Canada Western Red Spring Wheat. Cereal Chem. J..

[12] Hucl P., Briggs C., Shirtliffe S., Beres B., Spaner D., Dyck A., Gerard G. (2022). Increasing grain yield while maintaining baking quality in Canada Western Red Spring wheat. Can. J. Plant Sci..

[13] Kosová K., Chrpová J., Šíp V. (2009). Cereal Resistance to Fusarium Head Blight and Possibilities of its Improvement through Breeding. Czech J. Genet. Plant Breed..

[14] Tshikunde N.M., Mashilo J., Shimelis H., Odindo A. (2019). Agronomic and Physiological Traits, and Associated Quantitative Trait Loci (QTL) Affecting Yield Response in Wheat (*Triticum aestivum* L.). Front. Plant Sci..

[15] Beche E., Benin G., da Silva C.L., Munaro L.B., Marchese J.A. (2014). Genetic Gain in Yield and Changes Associated with Physiological Traits in Brazilian Wheat during the 20th Century. Eur. J. Agron..

[16] Graybosch R.A., Peterson C.J. (2010). Genetic Improvement in Winter Wheat Yields in the Great Plains of North America, 1959–2008. Crop Sci..

[17] Schauberger B., Ben-Ari T., Makowski D., Kato T., Kato H., Ciais P. (2018). Yield Trends, Variability and Stagnation Analysis of Major Crops in France over More than a Century. Sci. Rep..

[18] Kim S.H., Vujanovic V. (2016). Relationship between Mycoparasites Lifestyles and Biocontrol Behaviors against *Fusarium* Spp. and Mycotoxins Production. App. Microbiol. Biotechnol..

[19] Vujanovic V., Islam M.N., Daida P. (2019). Transgenerational role of seed mycobiome—An endosymbiotic fungal composition as a prerequisite to stress resilience and adaptive phenotypes in *Triticum*. Sci. Rep..

[20] Vujanovic V. (2021). *Tremellomycetes* Yeasts in Kernel Ecological Niche: Early Indicators of Enhanced Competitiveness of Endophytic and Mycoparasitic Symbionts against Wheat Pathobiota. Plants.

[21] Vujanovic V., Goh J.K. (2009). *Sphaerodes mycoparasitica* sp. nov., a new biotrophic mycoparasite on *Fusarium avenaceum*, *F. graminearum* and *F. oxysporum*. Mycol. Res..

[22] Kim S.H., Lahlali R., Karunakaran S., Vujanovic V. (2021). Specific mycoparasite-*Fusarium graminearum* molecular signatures in germinating seeds disabled Fusarium Head Blight pathogen’s infection. Int. J. Mol. Sci..

[23] Vujanovic V., Goh Y.K. (2012). qPCR Quantification of *Sphaerodes mycoparasitica* biotrophic mycoparasite interaction with *Fusarium graminearum*: In vitro and in planta Assays. Arch. Microbiol..

[24] Kim S., Vujanovic V. (2017). Biodegradation and biodetoxification of *Fusarium* mycotoxins by Sphaerodes mycoparasitica. AMB Exp..

[25] Goh Y.K. (2010). Molecular and Microscopic Studies of a Fusarium-Associated Biotrophic Mycoparasite. Master’s Thesis.

[26] Vujanovic V., Kim S.H. (2017). Adaptability of Mitosporic Stage in Sphaerodes Mycoparasitica towards Its Mycoparasitic-Polyphagous Lifestyle. Mycologia.

[27] Deng Z., Cui Y., Han Q., Fang W., Li J., Tian J. (2017). Discovery of Consistent QTLs of Wheat Spike-Related Traits under Nitrogen Treatment at Different Developmenttages. Front. Plant Sci..

[28] Gurevich A., Saveliev V., Vyahhi N., Tesler G. (2013). QUAST: Quality Assessment Tool for Genome Assemblies. Bioinformatics.

[29] Haidukowski M., Pascale M., Perrone G., Pancaldi D., Campagna C., Visconti A. (2005). Effect of Fungicides on the Development of Fusarium Head Blight, Yield and Deoxynivalenol Accumulation in Wheat Inoculated under Field Conditions with Fusarium Graminearum and Fusarium Culmorum. J. Sci. Food Agric..

[30] Duan Y., Xiao X., Li T., Chen W., Wang J., Fraaije B.A., Zhou M. (2018). Impact of Epoxiconazole on Fusarium Head Blight Control, Grain Yield and Deoxynivalenol Accumulation in Wheat. Pestic. Biochem. Physiol..

[31] Gao L., Turner M.K., Chao S., Kolmer J., Anderson J.A. (2016). Genome Wide Association Study of Seedling and Adult Plant Leaf Rust Resistance in Elite Spring Wheat Breeding Lines. PLoS ONE.

[32] Caldwell C.D., MacDonald D., Jiang Y., Cheema M.A., Li J. (2017). Effect of fungicide combinations for Fusarium head blight control on disease incidence, grain yield, and quality of winter wheat, spring wheat, and barley. Can. J. Plant Sci..

[33] Berry P.M., Kendall S., Rutterford Z., Orford S., Griffiths S. (2015). Historical Analysis of the Effects of Breeding on the Height of Winter Wheat (*Triticum aestivum*) and Consequences for Lodging. Euphytica.

[34] Martens G., Lamari L., Grieger A., Gulden R.H., McCallum B. (2014). Comparative yield, disease resistance and response to fungicide for forty-five historic Canadian wheat cultivars. Can. J. Plant Sci..

[35] Bai G., Shaner G. (2004). Management and Resistance in Wheat and Barley to Fusarium Head Blight. Ann. Rev. Phytopathol..

[36] McMullen M., Bergstrom G., De Wolf E., Dill-Macky R., Hershman D., Shaner G., Van Sanford D. (2012). A Unified Effort to Fight an Enemy of Wheat and Barley: Fusarium Head Blight. Plant Dis..

[37] Niwa S., Kubo K., Lewis J., Kikuchi R., Alagu M., Ban T. (2014). Variations for Fusarium Head Blight Resistance Associated with Genomic Diversity in Different Sources of the Resistant Wheat Cultivar ‘Sumai 3’. Breed. Sci..

[38] Vujanovic V., Goh Y.K. (2011). *Sphaerodes mycoparasitica* Biotrophic Mycoparasite of 3-Acetyldeoxynivalenol- and 15-Acetyldeoxynivalenol-Producing Toxigenic *Fusarium graminearum* Chemotypes. FEMS Microbiol. Lett..

[39] Mesterházy A. (1995). Types and Components of Resistance to Fusarium Head Blight of Wheat. Plant Breed..

[40] Berraies S., Cuthbert R., Knox R., Singh A., DePauw R., Ruan Y., Bokore F., Henriquez M.A., Kumar S., Burt A. (2023). High-density genetic mapping of Fusarium head blight resistance and agronomic traits in spring wheat. Front. Plant Sci..

[41] Powell A.J., Kim S.H., Cordero J., Vujanovic V. (2023). Protocooperative Effect of *Sphaerodes mycoparasitica* Biocontrol and Crop Genotypes on FHB Mycotoxin Reduction in Bread and Durum Wheat Grains Intended for Human and Animal Consumption. Microorganisms.

[42] Op De Beeck M., Lievens B., Busschaert P., Declerck S., Vangronsveld J., Colpaert J.V. (2014). Comparison and Validation of Some ITS Primer Pairs Useful for Fungal Metabarcoding Studies. PLoS ONE.

[43] de la Cuesta-Zuluaga J., Escobar J.S. (2016). Considerations for Optimizing Microbiome Analysis Using a Marker Gene. Front. Nutr..

[44] SAS/STAT® (2024). 9.3 User’s Guide: The MIXED Procedure.

[45] SAS Institute Inc. (2012). User’s Guide: Statistics. Release 9.3.

[46] Chaiwong N., Rerkasem B., Pusadee T., Prom-u-thai C. (2021). Silicon application improves caryopsis development and yield in rice. J. Sci. Food Agric..

[47] Grabka R., d’Entremont T.W., Adams S.J., Walker A.K., Tanney J.B., Abbasi P.A., Ali S. (2022). Fungal Endophytes and Their Role in Agricultural Plant Protection against Pests and Pathogens. Plants.

